# Leishmaniasis: Recent epidemiological studies in the Middle East

**DOI:** 10.3389/fmicb.2022.1052478

**Published:** 2023-02-02

**Authors:** Chinyere A. Knight, David R. Harris, Shifaa O. Alshammari, Ayele Gugssa, Todd Young, Clarence M. Lee

**Affiliations:** ^1^Department of Biology, Tuskegee University, Tuskegee, AL, United States; ^2^Department of Biology, University of Hafr Al-Batin, Hafr Al-Batin, Saudi Arabia; ^3^Department of Biology, Howard University, Washington, DC, United States

**Keywords:** leishmaniasis, cutaneous, visceral, vector, Middle East, treatment

## Abstract

Leishmaniasis, one of the most neglected tropical diseases (NTDs), is the third most important vector-borne disease worldwide. This disease has a global impact and severity of the infection and is greatest in the Middle East. The agent of infection is a protozoan parasite of the genus, *Leishmania*, and is generally transmitted by blood-sucking female sandflies. In humans, there are three clinical forms of infection: (1) cutaneous (CL), (2) mucocutaneous (ML), and (3) visceral leishmaniasis (VL). This review aims to discuss the current epidemiological status of leishmaniasis in Saudi Arabia, Iraq, Syria, and Yemen with a consideration of treatment options. The elevated risk of leishmaniasis is influenced by the transmission of the disease across endemic countries into neighboring non-infected regions.

## Introduction

Leishmaniasis is a parasitic disease caused by an intracellular protozoan of the genus *Leishmania* with a huge impact on human health. *Leishmania* is a flagellated protozoan parasite of the order *Kinetoplastida* and the family *Trypanosomatidae* (Kaufer et al., 2017). Worldwide, over 12 million people are infected with *Leishmania* spp., and an additional 350 million are at risk of infection (Lockard et al., 2019). A high prevalence of leishmaniasis is found primarily among poor populations of the poorest countries impacted by underfunded elimination programs, little to no interest from pharmaceutical industries, and inadequate healthcare infrastructure (Choi et al., 2021; Oliìas-Molero et al., 2021; Scarpini et al., 2022). These factors contribute to the spread and proliferation of the disease. Overall, leishmaniasis is endemic to Asia, the Middle East, North Africa, East Africa, the Mediterranean, and South and Central America (Tabbabi, 2019). Leishmaniasis is caused by ≈20 *Leishmania* species carried by various phlebotomine sandfly species. The *Leishmania*-sandfly vector triad may be restrictive or permissive. Restrictive vectors are mediated by ligand-receptor interchange specific to the *Leishmania* species (i.e., *Phlebotomus papatasi* and *Phlebotomus sergenti*) transmitted. Permissive vectors permit distinct *Leishmania* species (i.e., *Phlebotomus arabicus* and *Lutzomyia longipalpis*) that develop within the sandfly midgut and disturb immune signal pathways (Cecílio et al., 2022).

The geographical distribution and seasonal preference of sandflies vary by species. In general, most species survive at temperatures ranging from 16 to 44°C. Optimal activity occurs on warm, clear nights with low wind speed. Morphologically, sandflies are no more than 3.5 mm in length and covered with dense hair, holding their wings in a characteristic V-shaped position. Female adults feed on blood as a meal for the development of egg batches (Doha and Samy, 2010; Maroli et al., 2013; Salam et al., 2014). A parasitic infection spread by the bite of female *Phlebotomus* sp. leads to the transmission of the disease in the old world including Asia, Africa, and Europe, while *Lutzomyia* leads to the transmission of the disease in the new world including the Americas (Abdellahi et al., 2022).

Leishmaniasis manifests in clinically distinct diseases emerging as cutaneous (CL), mucocutaneous (ML), or visceral leishmaniasis (VL) (Alvar et al., 2012; Salam et al., 2014). According to the World Health Organization (WHO), in 2020, VL caused more than 90% of the cases that occurred in seven countries: Brazil, Ethiopia, India, Kenya, Somalia, South Sudan, and Sudan. In 2016, CL caused more than 84% of the cases reported in 10 countries: Afghanistan, Algeria, Brazil, Colombia, Iraq, Pakistan, Peru, the Syrian Arab Republic, Tunisia, and Yemen (WHO, 2020b; Scarpini et al., 2022). In the Middle East, there are several different *Leishmania* parasites that cause leishmaniasis.

Cutaneous leishmaniasis, which is the most commonly reported form of the disease, is identified by ulcerative skin lesions. It is caused by *L. major*, transmitted through *P. papatasi*, with rodent species of *Psammomys obesus, Meriones libycus*, *Nesokia indica*, and *Rhombomys opimus* serving as non-human reservoirs (Rafati and Modabber, 2014; de Vries and Schallig, 2022). VL is the most severe form of the disease in which the pathogen disseminates to internal organs such as the liver, the spleen, and the bone marrow. It is caused by *L. infantum*, transmitted through *P. galilaeus, P. syriacus, P. tobbi, P. halepensis*, with *Canisfamiliaris* (the dog species) acting as a non-human reservoir. Anthroponotic CL (ACL) is caused by *L. tropica* and spreads through the vector *P. sergenti*, circulating exclusively in humans. In addition, the etiological agent of Anthroponotic VL (AVL) is *L. donovani* and is transmitted through the *P. alexandri* vector (Coleman et al., 2011; Jacobson, 2011; Ready, 2013).

### Methodology

In total, 12 searches conducted in PubMed were used to obtain references for this review. Pubmed is the search platform that enables access to MEDLINE and PubMed Central. MEDLINE is the central database for the United States National Library of Medicine (NLM), which catalogs international journal articles. PubMed Central (PMC) is another database, which catalogs journal articles funded by the United States National Institutes of Health (NIH), including journal articles, which meet NIH quality standards and any journal article whether or not cataloged in MEDLINE or PubMed Central (Misra and Ravindran, 2022).

Four searches were carried out using the terms “leishmaniasis Saudi Arabia” (which yielded 238 results), “leishmaniasis Iraq” (which yielded 149 results), “leishmaniasis Syria” (which yielded 134 results), and “leishmaniasis Yemen” (which yielded 42 results). All results yielded reflect the publication period from 2000 to 2022 queried in MEDLINE/PubMed. The searches were conducted in September 2022. Searches were restricted by country (Saudi Arabia, Iraq, Syria, and Yemen) when used in conjunction with the term “leishmaniasis.” There were no restrictions on language.

Four additional searches were carried out using the terms “leishmaniasis drug trial Saudi Arabia” (which yielded six results), “leishmaniasis drug trial Iraq” (which yielded four results), “leishmaniasis drug trial Syria” (which yielded one result), and “leishmaniasis drug trial Yemen” (which yielded two results). All results yielded reflect the publication period from 2000 to 2022 queried in MEDLINE/PubMed. As stated previously, the searches were conducted in September 2022 and were restricted by country (Saudi Arabia, Iraq, Syria, and Yemen) when used in conjunction with the term “leishmaniasis.”

The data in this review were gathered primarily from WHO reports for each country and from an extensive literature search on PubMed using the term “leishmaniasis” followed by the name of each Middle Eastern country. Similar searches were carried out using the terms “drug trial,” “incidence,” “neglected tropical disease leishmaniasis,” “sandfly,” and “leishmaniasis vaccine Middle East” (yielded 13 results for the period between 2020 and 2022). Ultimately, queries were filtered down to the publication period from 2010 to 2022 in MEDLINE/Pubmed and focused on the country area endemicity, epidemiology, drug trials, and treatment options.

### Host-pathogen transmission

The insect vector, the sandfly, has multiple species but distinct subsets (Steverding, 2017). *Leishmania* parasites are transmitted through the bites of infected female phlebotomine sandflies, within the genera *Phlebotomus* and *Lutzomyia* (Steverding, 2017). The infection is initiated when the sandfly attacks the host using its proboscis to pierce the skin, creating a small wound from which blood pools from injured capillaries. The infectious promastigote enters the sandfly’s foregut and replicates. Then, when the sandfly feeds on another host, the parasite is transmitted through the injection of contaminated blood (Steverding, 2017; Maxfield and Crane, 2022). Once parasites gain access to the host, they are rapidly phagocytosed by macrophages directly or through apoptotic neutrophils through a complement receptor (CR3)-dependent mechanism. Inside macrophages, *Leishmania* sp. transforms into amastigotes and avoids degradation by *lipophosphoglycan* (LPG)-mediated disruption of lipid microdomains in the *phagosomal* membrane, thus impairing macrophage-based defensive mechanisms (Sehgal et al., 2012; Cecílio et al., 2022).

Moreover, inside the phagosome, *Leishmania* sp. produces a surface acid phosphatase, modulates intracellular phosphatases and kinases, and downregulates MHC II expression on the surface of infected macrophages (Yasmin et al., 2022). Together, these cellular events lead to unresponsive and infected macrophages, pro-inflammatory cytokine gene expression, and inhibition of leishmanicidal mechanisms, i.e., the production of reactive nitrogen and oxygen intermediates (Holowka and Bucala, 2020; Yasmin et al., 2022). Furthermore, *Leishmania* downregulates dendritic cell-based immune responses by inhibiting migration, maturation, antigen presentation abilities, and the production of IL-12 (Horta et al., 2012; Ehrlich et al., 2014, 2019). These effects on dendritic cells influence the development of a successful Th1 response required to clear the infection (Basit et al., 2022). Whereas infections with *L. major* result in self-healing lesions and lifelong immunity, infections with *L. donovani* lead to subclinical infection, resulting in protective immunity or, in clinical cases, fatal outcomes if not treated. The development of protective immunity appears to be dependent on the infecting species, host genetic factors, and the size of the inoculum (Horta et al., 2012; Ehrlich et al., 2014, 2019). The epidemiology of leishmaniasis depends on the characteristics of the parasite species, the local ecological characteristics of the transmission sites, current and past exposure of the human population to the parasite, and human behavior (WHO, 2020a). Currently, there is concern that the emergence of drug-resistant parasites emphasizes the need for a new treatment that can harness the host’s immune response. Therefore, effective control and treatment of leishmaniasis should take into account the influence of biological immune responses (Lockard et al., 2019).

### Epidemiology of leishmaniasis in selected Middle Eastern countries

#### Saudi Arabia

**FIGURE 1:**
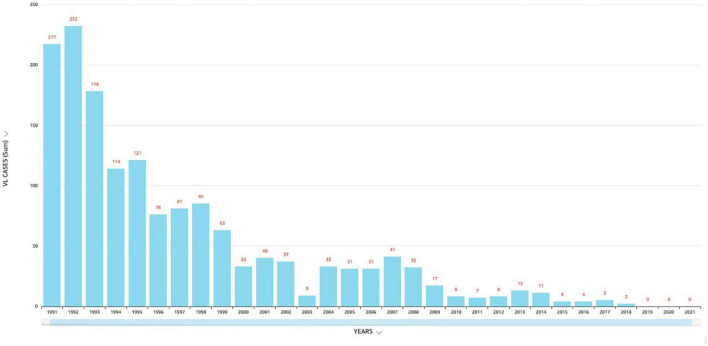
Number of visceral leishmaniasis cases reported in Saudi Arabia (1991–2021).

**FIGURE 2:**
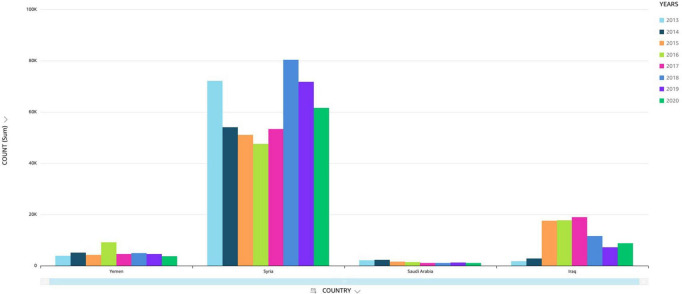
Cases of cutaneous leishmaniasis reported (2013–2020).

**FIGURE 3:**
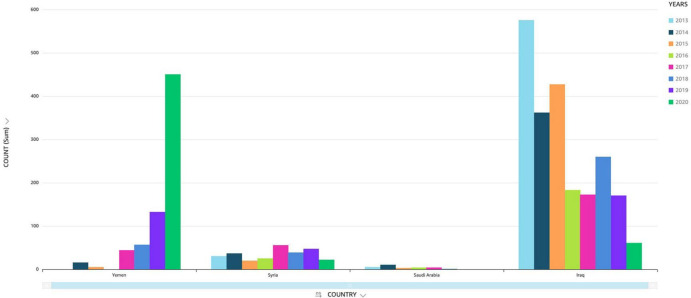
Cases of visceral leishmaniasis reported (2013–2020).

The Kingdom of Saudi Arabia (KSA) is situated in Southwest Asia with an estimated land space of 2,217,949 square km with different ecological and climatic patterns (Abuzaid et al., 2017, 2020). In general, (CL) is the most common form of the disease present in Saudi Arabia and is commonly known as *dommal*, *nafra*, and *El-mohtafara* (Salam et al., 2014). The (KSA) first reported (CL) in the 17th century. According to the Saudi Arabia Ministry of Health, the number of reported (CL) cases dropped from thousands in the 1980’s and 90’s to ≈600 in 2021. While the number of (VL) cases dropped from 217 in 1991 to zero in 2019, 2020 and 2021 (Abuzaid et al., 2022). For comparison, the number of (VL) cases in Yemen increased (see Figures 1–3). Saudi Arabia’s leishmaniasis control program (LCP) is based on a healthcare management system that includes health-care-data tracking all thirteen administrative provincial regions (Makkah, Madinah, Riyadh, Qasim, Ha’il, Jawf, Tabuk, Najran, Asir, Jizan, Bahah, the Northern and Eastern Province). In addition, pentavalent antimonials (sodium stibogluconate and meglumine antimoniate) and AmB (liposomal amphotericin B) are readily available and used for (VL) treatment. Also, drug treatment services are available for lengthy dosages that require complex in-patient administration and follow-up procedures (Al-Bajalan et al., 2018). Currently, Saudi Arabia is not in the top 10 of highest endemic countries, but is still the fourth most endemic area in western Asia (Alvar et al., 2012; Abuzaid et al., 2017, 2020, Al-Bajalan et al., 2018). (CL) continues to be a significant public health concern in the (KSA) with cases of (CL) in endemic regions which challenge the national health authorities.

Several factors in Saudi Arabia have led to the emergence of CL including (1) rapid urbanization, (2) the development of agriculture, (3) climate change, and (4) human migration. (Abuzaid et al., 2017, 2020). Particularly, the immigration of non-immune individuals into endemic areas is a factor in the emergence of leishmaniasis (WHO, 2020b). Moreover, millions of visitors to the holy places of Makkah and Madinah arrive for the Hajj pilgrimage every year and throughout the year for Umra (Abuzaid et al., 2017, 2020). The area under climate change impacts the distribution of the disease by influencing vector survival, parasite development, and the host’s movement. In Saudi Arabia, the vast majority of CL cases are reported from six endemic regions in Al-Qaseem and Riyadh (Central), Al-Hassa (East), Aseer (South West), and Ha’il and Al-Madinah (North West) (Abuzaid et al., 2017, 2020). Previous data reported that the highest numbers of cases are registered in the regions of Ha’il (282 newly detected cases), followed by Al-Madinah (203 newly detected cases), and Al-Hassa (196 newly detected cases) (Abuzaid et al., 2017, 2020; Rasheed et al., 2019). An epidemiological study, conducted in Ha’il from 2015 to 2016, reported that *Leishmania major* is the cause of CL in northwestern Saudi Arabia (Haouas et al., 2015, 2017).

The molecular detection of *Leishmania* DNA in *Ph. papatasi* and *Ph. kazeruni* supported the role of these two species in *Leishmania* transmission in the Ha’il and surrounding regions (Haouas et al., 2017). In Al-Madinah, the first comprehensive study of leishmaniasis, using the molecular characterization of CL, confirmed that *Leishmania major* and *Leishmania tropica* were the prevalent species in the Al-Madinah region (El-Beshbishy et al., 2013). Elmekki et al. (2017), studied CL in Al-Madinah city, during 2012–2015, and found that CL is prevalent in Al-Madinah and emerges into new areas, such as Almelelehy in the North, Yanbua’ in the West, Alhanakeia in the East, and Abar Ali in the South. Another investigation to identify the distribution of sandflies in the Al-Madinah and Asir regions of Saudi Arabia showed that no single restriction enzyme could separate species belonging to the same genera (like *P. papatasi* and *P. sergenti* by *Ase*I), as well as those belonging to different genera (like *P. papatasi* and *S. clydei* by *Ase*I), inducted that the genetic diversity within sandfly species based on the PCR–RFLP technique was non-specific (Al-Dakhil et al., 2017; Alraey, 2022).

A study in the Al-Taif region showed that the number of CL cases increased in the winter with localized skin lesions among young age, male gender, and rural residence (Hawash et al., 2018). In 2018, Sirdar et al. (2018) investigated the epidemiological trends of leishmaniasis in Jazan and Southern Saudi Arabia (Abdelhaleem et al., 2021). They found that both CL and VL were considered endemic diseases in the region. Moreover, this study showed that the species causing the disease is *Leishmania tropica*, with *Phlebotomus sergenti* being the main vector. *Leishmania donovani sensu lato* was the main VL parasite in the region; feral dogs are the reservoir, and *P. alexandri* and *P. orientalis are* suspected as main vectors (Sirdar et al., 2018). A molecular study detected human cases of anthroponotic *L. tropica* in Al-Ahsa, an eastern region in Saudi Arabia, for the first time (Al-Rashed et al., 2022). This may be accounted for by sandfly migration from regions outside Al-Asha during the COVID-19 lockdown, which caused vector-borne disease prevention programs and sandfly surveillance to shut down.

In summary, these data implicate *Leishmania major* and *Leishmania tropica* as key species in Saudi Arabia. In addition, CL and VL are considered endemic in the country. Although vaccination is yet to be developed, early and accurate diagnosis and treatment remain essential in the control of leishmaniasis in Saudi Arabia.

#### Iraq

Leishmaniasis is endemic in Iraq, where both cutaneous and visceral forms of the disease are reported. In Iraq, *Leishmania* spp. *infections* are common in the hot and dry border region between northern and central areas. Locally, cutaneous leishmaniasis (aka “Oriental sore” or “Baghdad boil”) is a slowly spreading inflammatory skin sore (Lesho et al., 2005). Several methods have been used to diagnose *Leishmania* parasites from skin lesions of human patients including histopathological examinations, direct smears, cultures, and serological tests (Al-Bajalan et al., 2018, 2021). However, few studies have been conducted using PCR to characterize *Leishmania* strains in human cutaneous lesions. Nevertheless, in the U.S. military base in Southern Iraq, a phylogenetic study investigated the prevalence of different *Leishmania* species in sandflies using molecular and phylogenetic analysis (Al-Bajalan et al., 2018, 2021). A study, from 2011 to 2013, conducted in all the provinces in Iraq showed that men are at higher risk for CL compared with women. The majority of cases were recorded among those in age groups 5–14 and 15–45 years, with the most cases from the lowland which has moderate annual rainfall and a high rural population (Al-Warid et al., 2017). Epidemiological, molecular, and phylogenetic studies from 2014 to 2017, using sequence analysis of the cytochrome *b* gene to determine the danger of an outbreak of CL in Iraq, showed that CL in the borderline area between northern and central Iraq was due to *L. major.* Results showed that the *L*. *major* strain in Iraq was closely related to the *L*. *major* MRHO/IR/75/ER strain in Iran (Al-Bajalan et al., 2018, 2021). One study reported that Diyala province had the highest rate of visceral and cutaneous leishmaniasis infection (21.91 and 13.85%) from 2014 to 2017. This was during military operations when most Diyala residents were displaced (Lehlewa et al., 2021).

The primary cohort of individuals infected with (VL) was 1–4 years that recorded the highest rate of 62.04% while the main age group of individuals infected with cutaneous leishmaniasis was 5–14 years, which recorded the highest rate of 37.81%. This epidemiological study provided data essential to plan strategies for parasitic disease control in Iraq (Ali et al., 2018). Modey et al. (2018) studied VL in the US soldiers who were deployed in Iraq (from 2015 to 2017) using enzyme-linked immunosorbent assay (ELISA), rk39 test strips, quantitative polymerase chain reaction (PCR), and interferon gamma release (IGRA) assays. They identified VL in 19.5% of Operation Iraqi Freedom (OIF) troops, while travel to northwest Iraq correlated with the infection (Mody et al., 2019; Ibiapina et al., 2022). Recent data reported by Iraqi researchers show attempts to characterize *Leishmania* species causing CL among Iraqi patients using sequence analysis of Internal Transcribed Spacer1 (ITS1) at Waist Province. Results revealed that the prevalence of cutaneous leishmaniasis was very high (83.3%) with a statistically significant association with sex; men were more prone (56.4%) to CL as compared to women (43.6%). The authors surmised that the war activities induced the expansion of CL and increased the incidence rate due to inadequate access to medical treatment in endemic areas (Ali et al., 2018). Most of the cases of leishmaniasis in Iraq were caused by the *Leishmania major.* In immunocompromised patients, the lesions heal spontaneously and leave indented scars.

Overall, the reported data show the potential risk of *Leishmania* parasites and their insect vectors in the free zones and surrounding countries, such as Saudi Arabia, Turkey, Jordan, and Iran. For treatment, sodium (Na) stibogluconate, a pentavalent antimony compound, is the lone drug licensed by the Iraqi Ministry of Health to treat CL (Lehlewa et al., 2021). Therefore, long-term studies are needed to assess treatment regimens for visceral and cutaneous leishmaniasis in order to improve the quality and standardization of care for these neglected diseases.

#### Syria

**FIGURE 4:**
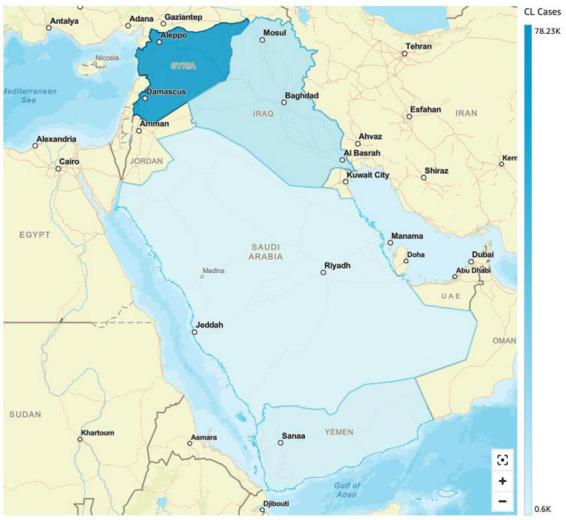
Incidence of cutaneous leishmaniasis (CL) in selected Middle Eastern countries (Saudi Arabia, Iraq, Syria and Yemen).

In 2019 alone, 89,357 cases of leishmaniasis were recorded in the Syrian Arab Republic. In 2018, the number of CL cases in Aleppo province was three times more when compared to 2007 (Muhjazi et al., 2019). The prevalence of *L. tropica* cases found in neighboring Lebanon was endemic to the species in nearby Aleppo and surrounding areas. This has much to do with the common name “Aleppo boil” for CL in Syria (Rehman et al., 2018). A wide-scale war offensive by the Islamic State (ISIS) in Aleppo led to a massive exodus of war refugees, a breakdown of public health infrastructure, and an accumulation of waste, generating breeding grounds for the vector *Phlebotomus serengeti* (carrier of *L. tropica*) (Muhjazi et al., 2019; Bizri et al., 2021). Overall, the factors that led to increased transmission of CL in Syria included the following: (1) migration of people, (2) poor housing, (3) non-immune people in endemic areas, (4) deficient medical facilities, (5) high rodent density, (6) unsanitary conditions, and (7) climate change (Al-Nahhas and Altawil, 2017). Previous data reported *L. tropica* as the predominant in northern Syria, causing nearly 80% of cutaneous lesions; while *L. major* was the second most prevalent, accounting for 13% of all *Leishmania* sp. (Al-Nahhas and Kaldas, 2013). In Syria, *L. infantum* was reported as the cause of CL in humans. However, *L. donovani* was isolated only from sandflies (Haddad et al., 2015). The World Health Organization’s statistics indicate that CL cases increased from ≈14,000 in 20,00 to ≈27,825 in 2010 (Rehman et al., 2018). Moreover, in early 2013, there was an alarming increase of CL to 41,000 cases (Al-Nahhas and Kaldas, 2013; Al-Salem et al., 2016). In January 2020 alone, ≈6178 cases of (CL) were reported. Most cases were reported from Deir-ez-Zor, Aleppo and Idleb regions (WHO, 2020b) (see Figure 4).

One of the recent studies aimed to characterize *Leishmania* sp. responsible for CL infections among Syrian refugees and compare them with recovered species/genotypes isolated from Jordanian patients. The results from this study indicated that 20 and 9 out of the inspected 66 patients (39 Jordanian and 27 Syrian) were infected with *L. major* and *L. tropica*, respectively. ITS1-PCR RFLP typing proved to be more sensitive in the detection of *Leishmania* species (positive in 44% of isolates) compared to both ITS1-5.8S rDNA gene and Kinetoplast DNA PCR, which were successful in identifying *Leishmania* species in 23 and 33% of the isolates, respectively. Moreover, sequencing and phylogenetic analysis of ITS1 and ITS1-5.8S rDNA genes revealed high levels of heterogeneity among sequenced isolates (Hijawi et al., 2019). These observations highlighted the need for further studies to confirm the strains, which may be introduced from Syria to neighboring countries (Hijawi et al., 2019).

In Syria, VL is far less common but shows increased mortality in infants and children. In recent years, thousands of cases have not been reported in Syria due to the absence of disease control programs. This creates opportunities for the disease to go unchecked. In addition, when Syrians travel long distances, they may carry *L. tropica* and *L. major* to the new communities. There is an increased risk of CL in non-infected neighboring countries.

#### Yemen

In 2019, 54.69% of people in the Republic of Yemen lived on < $1.50 USD per day, while 45.31% lived on $3.00-$5.50 USD per day (World Bank, 2021). Leishmaniasis in Yemen is not well recorded however, the Eastern Mediterranean Regional Office of the WHO gave Yemen a Red rating, which means priority action should be taken for (CL) and (VL) endemicity (Al-Kamel, 2016, 2017a,b; Warusavithana et al., 2022). Factors that may contribute to endemicity in Yemen include: 1) the complex geography, 2) weak infrastructure, 3) scarce medical facilities in rural areas, 4) deteriorating economic status, 5) high-cost healthcare and 6) ongoing war. The ongoing conflict has led to health system collapse and the safety of healthcare workers impedes patient treatment in war zones with travel challenges (Al-Kamel, 2016, 2017a,b; Gaber et al., 2022).

In 2013, an epidemiological study found that (CL), (ML) and (VL) are endemic to areas in Yemen where poverty and malnutrition play a significant role: (ML) was the most prevalent form (49.3%), followed by (CL) (47.4%), and (VL) (3.3%). In April-August 2013, the Regional Leishmaniasis Control Center reported (CL) (59%) in Al Bayda; the most endemic governorate (Al-Kamel, 2016, 2017a,b; Al-Daari et al., 2022). In 2016, the first entomological survey of sandfly fauna in northwestern Yemen reported *S. fallax* as the principal Sergentomyia genus and *S. dolichopa* as the least species encountered. Moreover, a possible zoonotic cutaneous transmission cycle due to Leishmania tropica in northwestern Yemen involved *P. arabicus* as the sandfly vector and the rock hyrax (procavia capensis) as the reservoir host (El-Sawaf et al., 2016). In Yemen, (CL) was reported in the northwestern, southwestern, and central highlands (Mahdy et al., 2016, 2010; Alkulaibi et al., 2019) and appears to be endemic in the northwestern highlands, Hajjah governorate region. In a previous study, *L. tropica*was reported as the main cause in about 95% of cases, while *L. donovani* and *L. infantum* were identified in 4.1% of cases (Alvar et al., 2012; Nassar et al., 2021).

Mahdy et al. characterized Leishmania sp. isolated from the bone marrow of Yemeni pediatric patients using sequence analysis of the ribosomal internal transcribed spacer-1 (ITS1) gene. Results indicated that ITS1 for microsatellite repeat numbers identified *L. infantum* in 11 isolates and *L. donovani* in 14 isolates. The data suggest the possibility of both anthroponotic and zoonotic transmission of (VL)-causing Leishmania sp. in Yemen (Mahdy et al., 2016). Asmaa et al. investigated the prevalence of (CL) in the Shara’b district, Taiz, using samples collected from 2012 to 2013. The results revealed that increases in serum malondialdehyde (*P* < 0.001) in (CL) patients and the highest level of MDA was related to overproduction of ROS and RNS resulting in oxidative stress and acceleration of lipid peroxidation in (CL) patients (Asmaa et al., 2017; Al-Harazi et al., 2021). Recently, the prevalence of (CL) in Utmah district located in the Western Highlands, found that (CL) was 18.5% and was more frequent in the escarpments with a prevalence of 37%, including 5.5% for active lesion and 31.5% for scar of healed lesions (Alkulaibi et al., 2019). Research revealed hundreds of thousands of individuals in Yemen with high potential of (CL) and (VL) infection in other parts of the country.

From January 2019 through June 2020, there were 6416 (CL) cases reported in Yemen. Most of the cases were from the mountainous Dhamar governorate. Males comprised 59%, while females comprised 41% of (CL) cases. Incidence rate of (CL) in Lahj governorate was 61.7/100,000 (per population), while the incidence rate in Aden, Al Mahra, and Socotra governorates were <1/100,000 (per population) (Al-Kamel, 2016, 2017a; Al-Daari et al., 2022).

### Drug development challenges

In the past century, ≈25 drugs were used for human leishmaniasis treatment. Antiparasitic drug discovery, in small part, was aided by public-funded sequencing of parasite genomes and the establishment of public-private partnerships (PPPs) with a focus on tropical diseases. The Medicines for Malaria Venture (MMV), the Institute for One World Health (IOWH), and the Drugs for Neglected Diseases (DNDi) are just a few PPPs involved in antiparasitic drug discovery (Nwaka and Ridley, 2003; Kettler and Marjanovic, 2004; Myler and Fasel, 2008).

The development of synthetic and natural drugs has shown equal importance in the quest for new therapeutics. Many infected people in rural areas cannot afford most modern drugs and rely on herbs to alleviate symptoms (Ghorbani and Farhoudi, 2017). Their medical use has been acknowledged by the Tropical Diseases Program of the World Health Organization (TDR/WHO) and the Drug Discovery Research Program (Chan and Pena, 2001; Fournet and Munoz, 2002). Overall, chemotherapy from herbal and synthesized anti-leishmanial agents provides few options to kill intracellular parasites. Some studies on herbal remedies illustrate positive *in vitro* and *in vivo* anti-leishmanial effects (Bekhit et al., 2018; Ilaghi et al., 2021; Hassan et al., 2022). Whereas other studies evaluating the antileishmanial activity of essential oils illustrate toxic effects in mammalian cells (Kingston and Newman, 2005; Hamdi et al., 2018; Cortes et al., 2020). Evidence-based clinical study trials of herbal remedies for treatment are scant. One of the few randomized clinical trials to assess the antileishmanial effect used *Juniperus excelsa* with cryotherapy on CL. All patients in the study groups were infected with *L. major* (Parvizi et al., 2017). In this study, 82% (27 out of 33 patients) had a complete cure after being administered *J. excelsa* M. Bieb (5%) hydroalcoholic leaf extract in conjunction with cryotherapy.

While the treatment for human leishmaniasis relies on chemotherapy, it is limited by drug resistance, adverse side effects, and high cost (Sunyoto et al., 2018; Salari et al., 2022). Current chemotherapies are often repurposed drugs (Barratt and Frail, 2012), i.e., pentavalent antimonials Sb (V), (sodium stibogluconate, glucantime, or meglumine antimoniate), amidine compounds (pentamidine), aminoglycosides (paromomycin), alkylphosphocholine compounds (miltefosine), and polyene antifungals (Amphotericin, AmB) (Dorlo et al., 2012; Oliìas-Molero et al., 2021; Pradhan et al., 2022) (see Supplementary Table 1).

Pentavalent antimonials [sodium stibogluconate (SSG) and meglumine antimoniate (MA)] are used to treat VL. However, there are myriad side effects that include cardiotoxicity, pancreatitis, nephrotoxicity, and hepatotoxicity. The toxicity mechanism is not well understood and antimonials have a short circulation time, whereby 80–95% is eliminated after 6–9 hrs. However, no association has been found between toxicity and Sb^III^ levels (Oliìas-Molero et al., 2021; Pradhan et al., 2022).

Pentamidine, when used against VL, shows side effects, i.e., cardiotoxicity, sudden decrease in blood pressure, and irreversible insulin-dependent diabetes mellitus. However, Pentamidine is mainly used against CL. It is used intravenously and is not available in oral drug formulation. It has the advantage of a short treatment, but efficacy varies for different *Leishmania* species, and its use may be associated with dysglycemia and other adverse effects (Oliìas-Molero et al., 2021; Pradhan et al., 2022).

Paramomycin’s (monomycin, aminosidine) ability to inhibit protein synthesis by discrete binding to 16S ribosomal RNA, low toxicity, and short treatment provides frontline antileishmanial use. However, resistances are easily created when used by themselves. It is obtained from *Streptomyces krestomuceticus* and produced by Pfizer. Paromomycin also happens to be a low-cost aminoglycoside antibiotic used to treat several parasitic agents (*Giardia*, *Entamoeba*) (Oliìas-Molero et al., 2021; Pradhan et al., 2022). Paromomycin has been used in combination with miltefosine as an alternative to treat patients in eastern Africa (Musa et al., 2022).

Miltefosine is used to treat VL and CL. It is given to patients in oral drug form (100–150 mg/day for 28 days). Adverse effects include a long half-life (>120 h), drug resistance, and abnormal fetal causation, which exclude miltefosine use in women during fertile life (Oliìas-Molero et al., 2021; Palić et al., 2022; Pradhan et al., 2022). Real and effective treatment for leishmaniasis remains elusive in large part because all leishmaniasis medicines derive from one or a tiny number of drug manufacturers. There remain key obstacles to accessing miltefosine, the only oral medicine approved for the treatment of leishmaniasis. Global availability of miltefosine was initially developed by a public–private partnership (PPP). It is now owned by Knight Therapeutics and represents a market failure since it depends on a single source, as no other quality-assured generic medicine is available on the market. The current price of miltefosine is three times higher than that in the initial PPP agreement, which is not affordable for the majority of the patients (Sunyoto et al., 2018). Challenges in access are further compounded by inefficient supply chains and unreliable forecasts. Many patients in Saudi Arabia, Iraq, Syria, and Yemen tend to be financially poor living in remote areas with low, next to no income (Choi et al., 2021). A phase II multicenter randomized study was done where, thermotherapy was combined with miltefosine for the treatment of (CL) (Lopez et al., 2022).

Amphotericin B (AmB) requires intravenous administration in a hospital setting. Patients are given 0.75–1 mg/kg per day for 25 days. However, due to adverse events such as renal toxicity, negative reaction to injection nephrotoxicity, and resultant kidney failure, these side effects initiated intensive research on new formulations (liposomes, emulsions, and nanoparticles), all aimed to reduce the amount of free AmB in the blood, minimize toxic concentrations, and transport the drug to infection location, efficiently. In 1997, liposomal solutions encapsulated with AmB were approved by the US Food and Drug Administration (FDA) for the treatment of VL (Meyerhoff, 1999). Liposomal AmB (Lip-AmB) is known commercially as ambisome, amphotec, and abelect. Recently, there are clinical studies on Lip-AmB, which only requires a single 3–5 mg/kg dose. However, a significant drawback is its high cost (Oliìas-Molero et al., 2021; Pradhan et al., 2022). Recently (AmB) has been formulated with PEGylated vesicles for (CL) treatment (Ramos et al., 2022).

Standard treatment for CL currently depends on multiple (biweekly) injections of antimoniate derivatives. Although antimoniate derivatives are the WHO-recommended treatment for CL, they present adverse effects, including site-injection pain, high cost, variable efficacy, and drug resistance. In Iran, a clinical pilot study was performed to assess the safety and efficacy of Lip-AmB (0.4%) in CL patients. All patients in the study groups were infected with *L. major.* Then, 12 out of 14 patients showed a complete cure with meglumine antimoniate/cryotherapy + Lip-AmB (0.4%) which is used twice a day for 28 days. In total, 18 out of 19 patients showed a complete cure with Lip-AmB (0.4%) which is used twice a day for 28 days. Only 15 out of 30 patients showed a complete cure with meglumine antimoniate with biweekly cryotherapy alone (Khamesipour et al., 2022). Studies with AmB in combination with miltefosine have shown positive results in treating VL (Diro et al., 2019; Burza et al., 2022).

Synthetic products have also shown great antileishmanial potential as inhibitors of methyltransferases (Magaraci et al., 2003) while alkyl-lysophospholipid derivatives are shown to have antileishmanial activities *in vitro* (Azzouz et al., 2005) and inhibitors of some III NAD-dependent deacetylase (Sereno et al., 2005). These compound classes have been considered somewhat advantageous including cost, novelty, retention time, and low intellectual property complications (Kingston and Newman, 2005) although they show high-level toxicity and lead to low patient selection for randomized clinical trials.

### Vaccine development challenges

Like drug development, vaccine development requires human clinical trials (I-IV). Phase I assesses primary vaccine safety and usually was conducted in <100 healthy volunteers. Phase II assesses the impact of multiple variables on immune responses and continues to assess the safety and side effects. It is typically conducted among 100–300 volunteers. Phase III is a large-scale clinical trial version of Phase II, albeit, conducted among 1000s of subjects in future routine-use conditions. Phase IV trials happen post FDA approval and are noted as post-marketing surveillance (PMS) trials.

Vaccination is more cost-effective than standard drug treatment in controlling VL and CL in humans. Vaccine design involves finding a suitable antigen and a suitable delivery system to induce an immune response. Using this principle, first-, second-, and third-generation antileishmanial vaccines have been developed, yet no patient candidates have had effective results for the transition to clinical trials beyond phase I. As a result, there is currently no licensed vaccine for human VL and CL (Malvolti et al., 2021; Tandon et al., 2023). The first-generation prophylactic vaccines for leishmaniasis were shown to be ineffective, despite their ability to induce Th1-type cytokine responses. The second-generation recombinant protein-based vaccines investigated preclinical animal models (mice, hamsters, and primates), but few reached phase I clinical trials. Recent clinical activity focused on adjuvant enhancement for use in combination with recombinant poly protein vaccines. The third-generation DNA vaccines encode one or more immunogenic proteins (either nucleic acids alone or as genes) and are introduced or added as delivery vectors. Furthermore known as viral vaccines, the third-generation DNA vaccines were recently used in a clinical trial, whereby the delivery route was selected to induce CD8+ T cells against leishmaniasis (Osman et al., 2017; Moafi et al., 2019).

The first-generation vaccines include studies on whole-killed parasites, fractionated *Leishmania* antigens, and live-attenuated pathogens. The whole-killed *Leishmania* vaccines have low costs and achieved limited success. For example, Leish-vaccine in humans was applied in Phase I and II clinical trials. However, this vaccine failed to achieve satisfactory results (Moafi et al., 2019). In Iran, different doses of inactivated *L. major* promastigotes with or without Bacillus Calmette–Gueìrin (BCG) were investigated in Phase I and II clinical trials for immunogenicity and safety. The results were negative (Ghorbani and Farhoudi, 2017; Moafi et al., 2019). Fractionated *Leishmania* antigens and live-attenuated pathogens are beyond the scope of this review because the studies show vaccine development for canines.

The second-generation vaccines include studies on recombinant proteins (LEISH-F1, LEISH-F2, and LEISH-F3). All three proteins were produced by the Infectious Disease Research Institute (IDRI, Seattle, WA, USA). LEISH-F1 reached Phase II of clinical trials. This artificial protein was encoded by three genes: *L. major* homolog of eukaryotic thiol-specific antioxidant (TSA), *L. major* stress-inducible protein-1 (LmSTI1), and *L. braziliensis* elongation and initiation factor (LeIF). In a different study, IDRI produced LEISH-F2 (Gillespie et al., 2016). This protein excludes the N-terminal histidine tag, resulting in more resemblance to natural proteins of wild species (Gillespie et al., 2016). Due to the substitution of glutamine for Lys274, the assembly process of LEISH-F2 was improved, compared with LEISH-F1 (Gillespie et al., 2016). After safety and immunogenicity approval, the vaccine entered Phase II clinical trial, where its therapeutic effects on CL patients were assessed and compared with chemotherapy (Gillespie et al., 2016). For this aim, LEISH-F2 (10 μg) was associated with MPL-SE adjuvant (25 μg) and the clinical cure period was determined for each patient. LEISH-F3 was another multicomponent vaccine comprized of two proteins: nucleoside hydrolase (NH) and sterol 24-c-methyltransferase (SMT), derived from *L. donovani* and *L. infantum*, respectively (Moreira et al., 2015; Gillespie et al., 2016). The application of the vaccine in healthy and adult individuals, living in Washington (U.S.), showed promising results as a robust immune response against VL (Chakravarty et al., 2011; Moafi et al., 2019). None of these experimental vaccines for leishmaniasis went further in clinical trials and were not fit for human use.

The third-generation vaccines include studies on DNA vaccines, which promote both cellular and humoral immunity. DNA vaccines are made up of heterologous DNA (usually a plasmid) that synthesizes antigenic proteins that produce specific immune responses. These DNAs are supplied by vectors that allow them to be expressed in eukaryotic cells (Liu et al., 2006; Abdellahi et al., 2022). By documenting the beneficial role of CD8+ T cells in the treatment and prevention of VL and PKDL (Post Kala-azar dermal leishmaniasis), many bodies of research have been focusing on the DNA vaccines (Osman et al., 2017). Recently, it was shown that a third-generation vaccine, employing semian adenovirus (ChAd63) could effectively elicit a wide range of CD8+ T cells, specified for *Leishmania* antigens (Osman et al., 2017). This vaccine encoded the KH gene, constituted of two genes of *L. donovani* antigens: KMP-11 and HASPB (Osman et al., 2017). The results of the study showed that not only intramuscular doses (1 × 1,010 and 7.5 × 1,010 ChAd63-KH) of ChAd63-KH were safe but also efficiently induced interferon-gamma production and dendritic cell activation (Myler and Fasel, 2008; Osman et al., 2017). As a result, the application of the ChAd63-KH vaccine was a promising approach for the prevention and treatment of *L. donovani* infection (Osman et al., 2017). The therapeutic effects of ChAd63-KH have been evaluated in Phase II of a non-randomized trial. However, there is no indication that this vaccine has moved on to Phase III clinical trials.

DNA vaccines offer several advantages which include: (1) fast, simple, and cheap large-scale production, (2) zero need for low temperature, transportation, and storage, and (3) the capacity to provide long-term protection against multiple strains of *Leishmania*. The main concern with these vaccines is the risk of parasite DNA entering the mammalian genome, which carries cancer risk and autoimmune disease (Liu et al., 2006; Abdellahi et al., 2022).

Leishmaniasis is the only human parasitic disease where vaccination works through a procedure known as leishmanization. It was used for decades in the Middle East (Pacheco-Fernandez et al., 2021). In this procedure, live and active *L. major* promastigotes are injected intradermally into the triangular-shaped deltoid muscle. An active ulcer then develops and eventually heals on its own. This results in long-term immunity against CL and provides cross-protection against VL (Osman et al., 2017). Despite the danger, it is still used in endemic areas among patients in Afghanistan, Iraq, and Iran. Leishmanization was used during the Iran–Iraq war but after that time it stopped due to a lack of safety (Khamesipour et al., 2005). Recently, *Leishmania* strains may be attenuated by genome-editing techniques involving CRISPR. This approach offers safer ways to induce long-term immunity and should make way for second-generation leishmanization in clinical trials (Pacheco-Fernandez et al., 2021).

Since multiple forms of leishmaniasis exist inside and outside the Middle East, a pan-*Leishmania* vaccine is needed despite the geographic infection location (Volpedo et al., 2021). Effective leishmanial vaccine development continues to be hindered by the global procurement landscape. Leishmaniasis is a small and unattractive market for pharmaceutical industries. They continue to show little to no interest in leishmaniasis elimination solutions. Furthermore, control programs are severely underfunded (Choi et al., 2021; Chang et al., 2022). One study suggested leishmaniasis vaccine development should have been as urgent as developing vaccines for COVID-19 (Chang et al., 2022).

## Conclusion

In addition to drug and vaccine development challenges, many patients and physicians resort to alternative therapies including animal-toxins, insecticide nets, immuno-therapy, photo-dynamic therapy and cryo-therapy alone or in-combination with synthetic drug therapies and leishmanization (Singh and Sivakumar, 2004; Parvizi et al., 2017; Garza-Tovar et al., 2020; Sridharan and Sivaramakrishnan, 2021).

Finally, there remains one solution, central to all the epidemiological studies discussed in this review; access to quality-assured medical resources (ie. pharmaceuticals, infrastructure, and technology). It is evident that leishmaniasis medicine global procurement contains significant disparities in supply versus demand among the top ten endemic countries, which include Iraq, Syria and Yemen (Choi et al., 2021). In Iraq (between 2013-2017), there were only <20,000 (CL) drug treatments delivered versus >50,000 (CL) cases. In Syria there were only <125,000 (CL) drug treatments delivered versus >225,000 (CL) cases. In Yemen the number of (CL) drug treatments delivered was in close proximity versus the number of (CL) cases reported. (Choi et al., 2021).

## Author contributions

All authors listed have made a substantial, direct, and intellectual contribution to the work, and approved it for publication.

## Supplementary material

Supplementary Table 1 Drug categories and options for controlling leishmaniasis.
