# Supplementary material for: Leishmaniasis: Recent epidemiological studies in the Middle East
Source: Front Microbiol. 2023 Feb 2;13:1052478. doi: 10.3389/fmicb.2022.1052478 (PMC9932337; doi:10.3389/fmicb.2022.1052478)
Supplement: Supplementary Table 1 — Drug categories and options for controlling leishmaniasis. [file Table_1.pdf]

CLASS OF DRUGS	TYPES
PENTAVALENT COMPOUNDS	Sb(V), Sodium Stibogluconate, Glucantime, or Meglumine Antimoniate
AMIDINE COMPOUNDS	Pentamidine
AMINOGLYCOSIDES	Paramomycin
ALKYLPHOSPHOCHOLINE COMPOUNDS	Miltefosine
POLYENE ANTIFUNGALS	Amphoteracin (AmB), Liposomal AmB (Lip-AmB, LAmB)

VACCINES	TYPES
1st GENERATION ANTILEISHMANIAL VACCINES	Whole killed parasites. Fractionated *Leishmania* antigen, Live attenuated pathogens
2nd GENERATION ANTILEISHMANIAL VACCINES	Recombinant Proteins; which are produced through Genetically Engineered Cells
3rd GENERATION ANTILEISHMANIAL VACCINES	Genetic Immunization; consist of nucleic acids or genes added into delivery vectors

THERAPIES	TYPES
ANIMAL-TOXIN THERAPY	Snake-derived venom from complex mixtures of peptides; crotoxin, gyroxin and PA2
CRYO-THERAPY	Liquid nitrogen at –196°C applied directly to the lesion, reaching lethal temperature
HERBAL THERAPY	Secondary metabolite extracts derived from roots, stalks, leaves, fruits, and seeds
IMMUNO-THERAPY	Vaccines, interferons (IFNs) and protein immunomodulators, or combination of these
PHOTODYNAMIC THERAPY	Topical ALA / MAL, followed by laser or IPL; kills host cells and thus kills parasites
THERMO-THERAPY	Heat application (50℃ for 30 seconds) usually requires the use of local anaesthesia
