# Supplementary figures and images for: Leishmaniasis: Recent epidemiological studies in the Middle East
Source: Front Microbiol. 2023 Feb 2;13:1052478. doi: 10.3389/fmicb.2022.1052478 (PMC9932337; doi:10.3389/fmicb.2022.1052478)

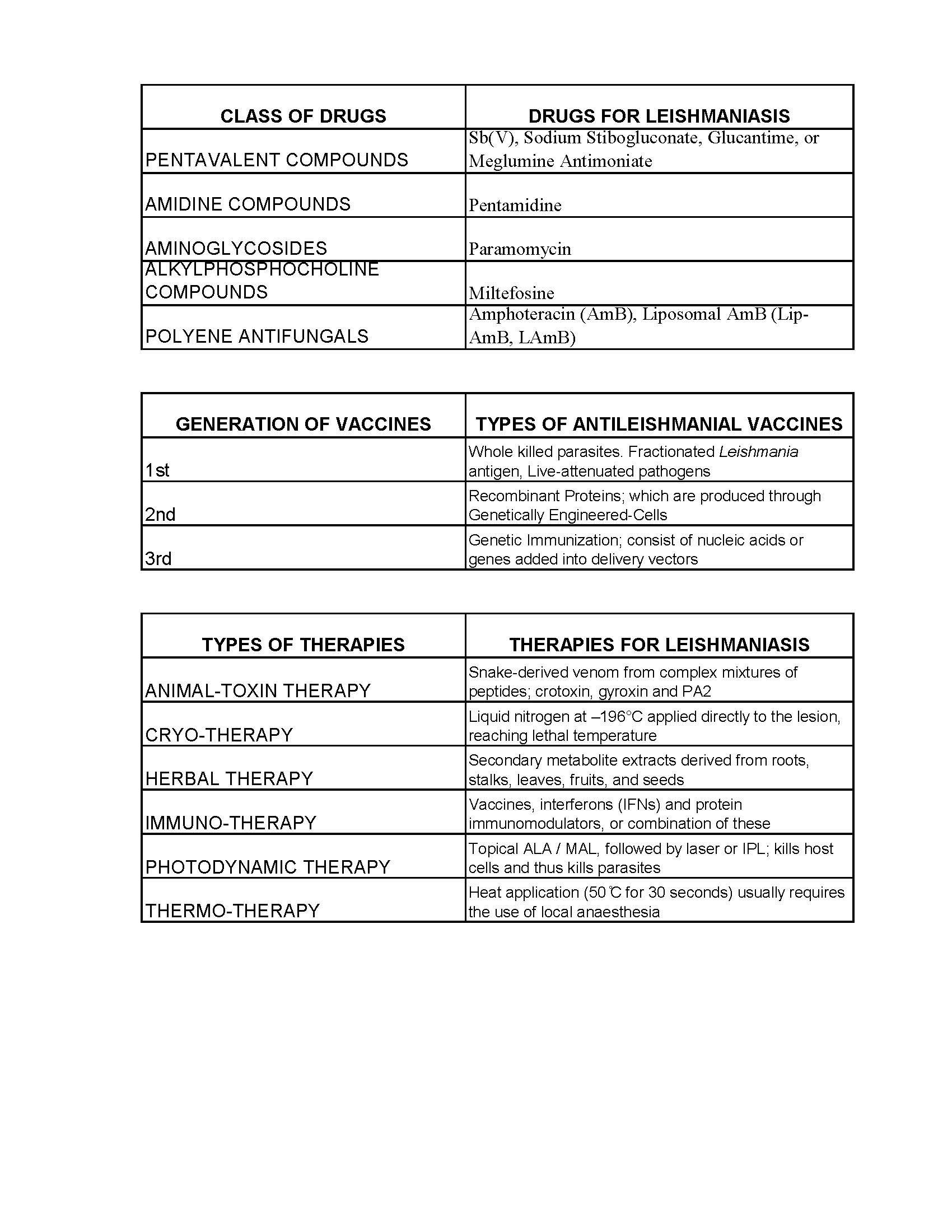

Supplement: Supplementary file 2 [file Image_1.JPEG]
